# Compressive response and buckling of graphene nanoribbons

**DOI:** 10.1038/s41598-018-27808-0

**Published:** 2018-06-25

**Authors:** A. P. Sgouros, G. Kalosakas, K. Papagelis, C. Galiotis

**Affiliations:** 1 School of Chemical Engineering, National Technical University of Athens (NTUA), GR-15780 Athens, Greece; 2 Institute of Chemical Engineering Sciences - Foundation of Research and Technology Hellas (FORTH/ICE-HT), GR-26504 Patras, Greece; 3 Department of Materials Science, University of Patras, GR-26504 Patras, Greece; 4 Crete Center for Quantum Complexity and Nanotechnology (CCQCN), Physics Department, University of Crete, GR-71003 Heraklion, Greece; 5 School of Physics Department of Solid State Physics, Aristotle University of Thessaloniki, GR-54124 Thessaloniki, Greece; 6 School of Chemical Engineering, University of Patras, GR-26504 Patras, Greece

## Abstract

We examine the mechanical response of single layer graphene nanoribbons (GNR) under constant compressive loads through molecular dynamics simulations. Compressive stress-strain curves are presented for GNRs of various lengths and widths. The dependence of GNR’s buckling resistance on its size, aspect ratio, and chiral angle is discussed and approximate corresponding relations are provided. A single master curve describing the dependence of the critical buckling stress of GNRs on their aspect ratio is presented. Our findings were compared to the continuum elasticity theories for wide plates and wide columns. In the large width limit, the response of the GNRs agrees with the predictions of the wide plates theory and thus, with that of wide graphenes. In the small width limit, the behavior of graphene nanoribbons deviates from that of periodic graphenes due to various edge related effects which govern the stiffness and the stability of the graphene membranes, but it qualitatively agrees with the theory of wide columns. In order to assess the effect of thermal fluctuations on the critical buckling stress a wide range of temperatures is examined. The findings of the current study could provide important insights regarding the feasibility and the evaluation of the performance of graphene-based devices.

## Introduction

Graphene constitutes the first truly two dimensional (2D) material^1,2^, characterized by record high mechanical^3–6^, thermal^7,8^ and electronic properties^1,9^. Due to its excellent properties graphene can give rise to a plethora of potential applications such as energy storage^10^, drug delivery^11^, metamaterials^12,13^, sensors^14–16^ and many more. Furthermore, graphene’s high Young modulus (E ~ 1 TPa) and tensile intrinsic strength (~130 GPa)^3^ makes it very attractive in applications regarding stretchable transparent electronics^9^ and as a reinforcing agent in nanocomposite materials^17,18^. Graphene strength may be further increased by charge doping^19^. However, for such applications the stress transfer efficiency and graphene’s ability to carry tensile and compressive loads is of outermost importance since it dictates the reliability, and the range of operation of potential devices.

The architecture of graphene membranes can be tailored either through strain engineering^20,21^ or through the introduction of localized defects^22–27^, leading to controllable construction of complex nanostructures such as nanoscrolls^22,25^ and nanocages^23,26^. Graphene, and other 2D materials discovered recently are characterized by unique mechanical properties. Upon crumpling, either through introducing local defects^27^, or through deformations by attachment to pre-stretched substrates^20^, graphene membranes present auxetic behavior and hence display negative Poisson ratio. Furthermore, as shown in ref.^28^ even pristine graphene displays auxetic behavior in the large tensile strain regime.

The precise conditions in which the graphene buckles under compressive loads are very important for assessing the feasibility and performance of graphene-based devices and thus, they have been studied extensively in the literature both experimentally^29–34^ and theoretically through studies involving quantum mechanical^35^, atomistic^36–41^ and continuum^42,43^ approaches. The nature of the substrate and the strength of the interface have been shown to have a dramatic effect on the stability of supported membranes under compression. The critical buckling strain of supported graphenes is larger by several orders of magnitude than the one of suspended graphenes as has been shown by experiments^29,32,44^ and a simulation study^45^.

Several numerical investigations of suspended graphenes^36–39^ have shown that the critical buckling stress of graphene presents an inverse square length dependence, in accordance with the Euler buckling of the linear elasticity theory of loaded slabs^46,47^. This holds irrespectively from the chiral angle^48^ of the loading direction^36,38,39^. However, it has been shown^30^ that compressive loads can increase the stress-transfer efficiency among the polymer matrix and the additives in graphene/epoxy nanocomposites, leading to slight under-predictions of the critical buckling stress by the Euler buckling formula. Conventional materials, as well as 2D materials with a thickness of just a few atoms, exhibit compressive (tensile) strain along the concave (convex) side when buckled^46,49^. In contrast, one atom thick 2D materials such as graphene are subjected to tensile deformation when buckled since the C-C bonds are elongated^39^.

Finite size effects on graphene nanoribbons (GNRs) as well as the structural features of their edges, can have a great impact on their electronic^50,51^, thermal^8^ and elastic^37,52,53^ properties. Due to the excess potential energy of the edge atoms, the edges of GNR perform significant in-plane and out-of-plane displacements in order to relax the edge forces^52^. GNRs with very small aspect ratios have been shown to be unstable, since they twist and bend spontaneously; this effect becomes stronger with increasing temperature^40^. Furthermore, due to the structural features of the edges, GNRs can display auxetic behavior since the tensile forces can smoothen the out of plane displacements of the edges, thus presenting negative Poisson ratio^53^.

In the present study, we investigate the behavior of single layer graphene nanoribbons with free edges (laterally unconstrained) under constant compressive loads, and examine various size and temperature effects on their resistance to buckle. The main aim is to provide qualitative comparisons among the examined GNRs and establish links between their response and the corresponding dimensions and aspect ratios. The length, width, and chiral angle dependence of the critical buckling stress as well as various effects related to the free edges are examined in depth while we present the temperature dependence of the critical buckling stress of GNRs as well. The corresponding variation on GNR’s width at different temperatures for nanoribbons with free edges is compared to that of graphene sheets with periodic edges at the lateral dimension^39^.

## Results and Discussion

### Compressive stress – strain curves

**Figure 1:**
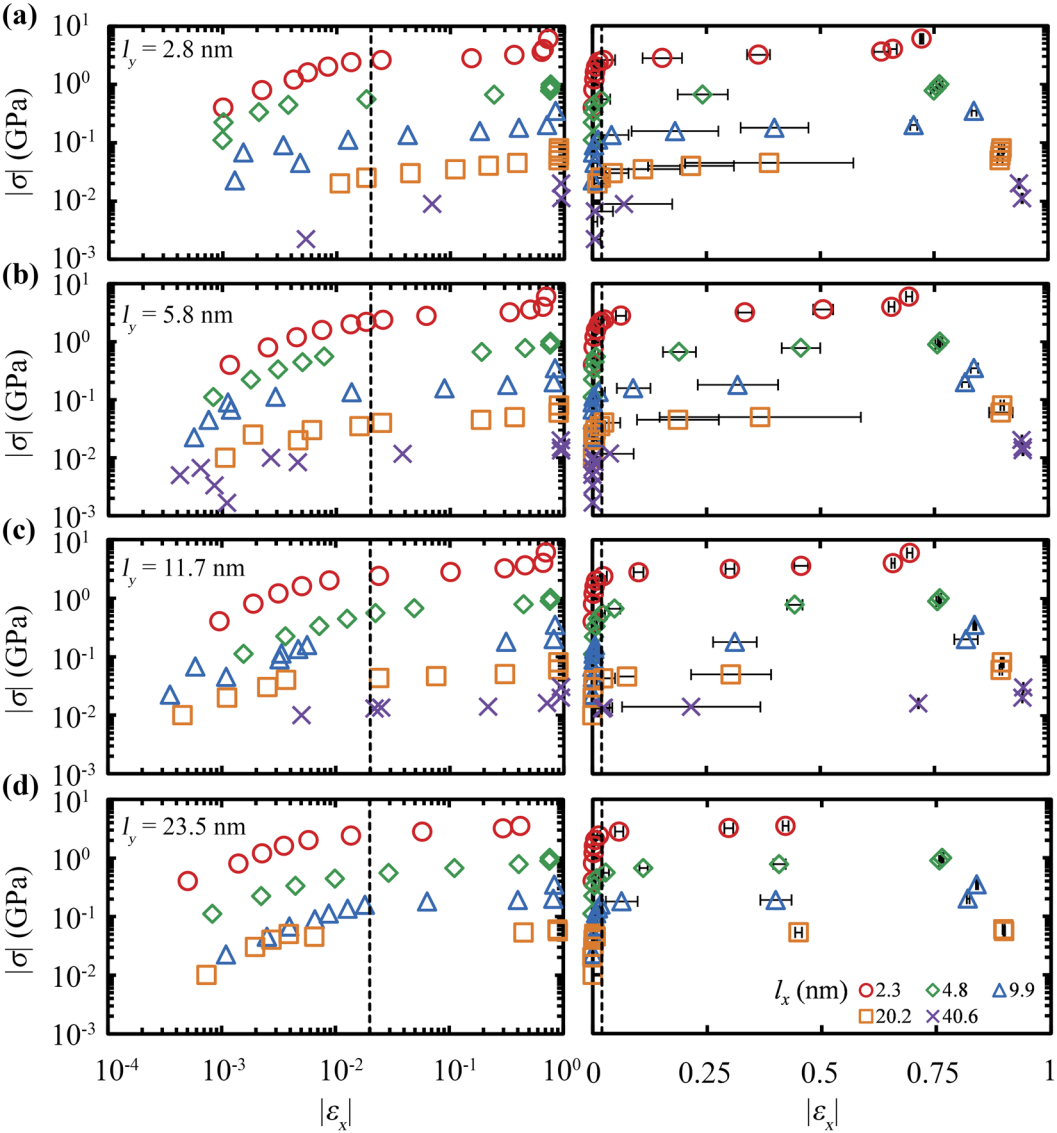
Compressive stress-strain curves of graphene ribbons with dimensions along the loading direction *l*_*x*_ = 2.3 (○), 4.8 (◊), 9.9 (△), 20.2 (□) and 40.6 (×) nm, and lateral dimensions (**a**) *l*_*y*_ = 2.8, (**b**) 5.8, (**c**) 11.7 and (**d**) 23.5 nm, at *T* = 300 K. The vertical dashed line marks the average critical buckling strain over all the studied samples, ${\bar{\varepsilon }}_{{\rm{crit}}}$ = 0.022 ± 0.002. For clarity, the stress-strain plots are presented in logarithmic (left) and semi logarithmic plots (right). Error bars depict the standard deviation from the mean strain.

Figure 1 displays compressive stress-strain curves for graphene ribbons of various sizes, at room temperature (*T* = 300 K). In these stress-controlled simulations, the strain is computed using the time averaged distance between the center of mass of the clamped regions. Irrespectively of the sheet dimensions, the compressive stress-strain curves display qualitatively similar behavior which can be classified into three main regimes: the elastic response, the plateau and the locking^54^ regime.

In particular, for a strain threshold up to ~2% the GNRs can carry compressive loads whose magnitude depends on their lengths and effectively resist buckling. After this strain threshold of about 2% the strain increases abruptly by slightly increasing stress (plateau regime)^55,56^ corresponding to the buckling of GNR. For even larger values of stress, however, the films fully collapse (locking regime) and the clamped regions at the opposite sides of the sheet come close together and repel each other due to repulsive nonbonded interactions. It should be noted that the deformation of the GNRs is not plastic, even at these very large strains, thus if the compressive loads are removed their initial dimensions will be restored.

Even though the response of the GNRs under compression seems qualitatively similar to that of pristine graphenes (when periodic boundary conditions on the lateral dimension are used)^39^, however, there are subtle differences due to the presence of the free edges. Edge atoms—being boundary defects—have higher potential energy with respect to the inner atoms^52^ since they take part in fewer covalent bonds and participate in fewer bond-bending angles and dihedrals; as a result they contribute much less to the flexural rigidity of the material. For example, an atom at the inner region of the GNR participates in 24 torsional angles, while an atom at a zigzag (ZZ) or an armchair (AC) edge participates in 10 or 9 angles, respectively. It has been shown in ref.^56^ that the dihedral angles contribute ~41% of the flexural rigidity of graphene, where the sheet was modeled by the second generation Brenner force field^57^.

**Figure 2:**
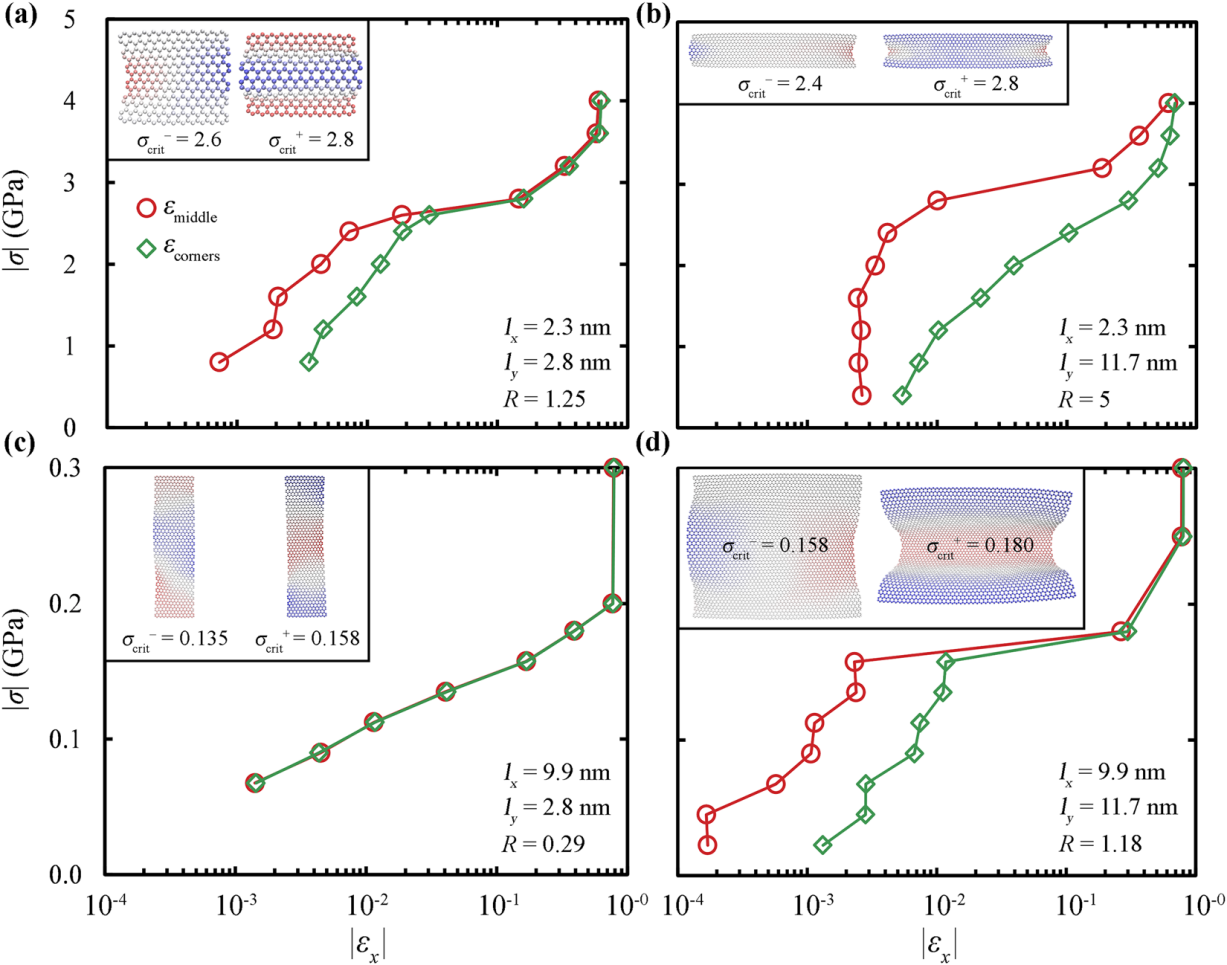
Compressive stress-strain curves extracted from the corners (◊) and from the middle (○) of the clamped graphene ends, for different aspect ratios, *R* = *l*_*y*_/*l*_*x*_, at room temperature. Lines are guides to the eye. The insets display atomistic representations of the sheets for buckling stresses just below (*σ*_crit_^−^) and just above (*σ*_crit_^+^) the critical buckling stress, while blue (red) colors illustrate positive (negative) displacements along *z*-axis.

Figure 2 shows the compressive stress-strain curves of GNRs of different aspect ratios *R* = *l*_*y*_/*l*_*x*_ and sizes, extracted from the middle (*ε*_middle_) and from the corners (*ε*_corners_) of the clamped regions; these are delimited by boxes with dots and dashes in Fig. 11, respectively. Apparently, the qualitative response of compressed GNRs is mainly governed by their aspect ratio and to a lower degree by their actual size. From Fig. 2 we see that:

1. For large aspect ratios (Fig. 2b), *ε*_corners_ > *ε*_middle_ before the locking regime, since the buckling of the corners is initiated at much smaller compressive stresses. For example, upon subjecting the GNR shown in Fig. 2b to 2.8 GPa the strain at the corners is about 30 times larger than the strain at the middle region of the ends.
2. In cases the aspect ratio is close to unity (Fig. 2a and d) the effect is much less pronounced since the deviation between *ε*_middle_ and *ε*_corners_ is observed only along the prebuckling regime. Note that the response of the sheets in Fig. 2a and d, which have similar aspect ratio but different dimensions, looks very similar with the exception that the plateau regime is broader in the larger sample.
3. Finally, for small aspect ratios (Fig. 2c) the response of the corners is near identical to that of the central region (*ε*_corners_ ≈ *ε*_middle_), since the distance between these regions is small for relatively small values of *l*_*y*_ and thus, the buckling of the corners and the middle region is synchronized.

Even in the absence of compressive stresses and geometric constrains, the edges of a GNR exhibit significant out-of-plane displacements, *w*. Such displacements cannot be attributed entirely to thermal fluctuations since they are also observed in the minimized structures of both clamped and free GNRs. Several minimized configurations of GNRs (using the Polak-Ribiere conjugate gradient algorithm^58^), modeled by the LCBOP^59^ and the TERSOFF^60,61^ force fields are presented in Section S2 of the supporting information (Figs S2–S5). Similar findings have been reported in refs^52,53,62^. The dimensions and the aspect ratio of the GNR and the strength of the imposed compressive stress have a strong effect on the shape and the stability of these displacements, which in many cases lead to the premature buckling of the edges, especially in GNRs with large aspect ratios.

For clamped GNRs, the out-of-plane displacements of the free edges exhibit a catenary-like shape, while the curvature, *d*^2^*w*/*dx*^2^, at the stationary points is either positive or negative and can be approximated by its discrete analogue due to the discrete nature of graphene. For low stress values the out-of-plane displacements of the free edges form metastable states since the sign of their curvature can spontaneously change with time. Interestingly, just before the buckling of the GNR, the curvatures of the opposing edges have usually opposite signs, i.e. the displacements of the two free edges are in the opposite sides of the GNR, as seen in the insets a, b and d of Fig. 2. For larger values of stress, however, the out-of-plane displacements of the free edges “lock” and thus the sign of the curvature does not change. In most cases, the opposing free edges bend along the same direction, as seen in Fig. 2, although in geometries with large aspect ratios there are cases where the free edges are being displaced in different sides of the sheet. In cases that there is a gradual loading of the GNR then the loading rate can affect this phenomenon. Some test simulations with finite loading rates (in this work the loading of the sheet is instantaneous) showed that when the large aspect-ratio GNRs are loaded gradually, their edges are more likely to buckle along the same side.

### Size dependence of the critical buckling stress

The transition from the elastic response to the plateau regime in the GNR’s compressive behavior can be quantified through the critical buckling stress, *σ*_crit_, which constitutes a threshold where small increases of stress lead to an abrupt increase of the strain, signifying the buckling of the sheet. Due to the presence of the free edges, this transition between the elastic and the plateau regime is in many cases much smoother compared to the one obtained by simulations of graphenes with periodic boundary conditions at the free of load edges^39^. One way to calculate the critical buckling stress in GNRs is to place it at the onset of the peak in the slope of the compressive strain-stress curves (*d*|*ε*|/*d*|*σ*|) calculated numerically by finite differences from consecutive data points. So, the critical buckling stress can be approximated as:

1 $${\sigma }_{{\rm{crit}}}=\frac{{\sigma }_{{\rm{crit}}}^{+}+{\sigma }_{{\rm{crit}}}^{-}}{2}$$

where ${\sigma }_{{\rm{crit}}}^{+}$ (${\sigma }_{{\rm{crit}}}^{-}$) is the recorded value of the stress just above (below) the aforementioned onset of the peak, while the critical buckling strain *ε*_crit_ is the recorded strain at ${\sigma }_{{\rm{crit}}}^{-}$ (due to the abrupt increase at higher loads). To quantify the error of *σ*_crit_ the following semi-difference formula was used:

2 $$\delta {\sigma }_{{\rm{crit}}}=\frac{{\sigma }_{{\rm{crit}}}^{+}-{\sigma }_{{\rm{crit}}}^{-}}{2}$$

Interestingly, *ε*_crit_ does not show significant dependence on *l*_*x*_ and *l*_*y*_, while the average *ε*_crit_ over all the examined samples equals to 0.022 ± 0.002. This coheres with the findings of ref.^32^ in which the estimated *ε*_crit_ was found to be insensitive to the aspect ratio of the examined embedded GNR.

**Figure 3:**
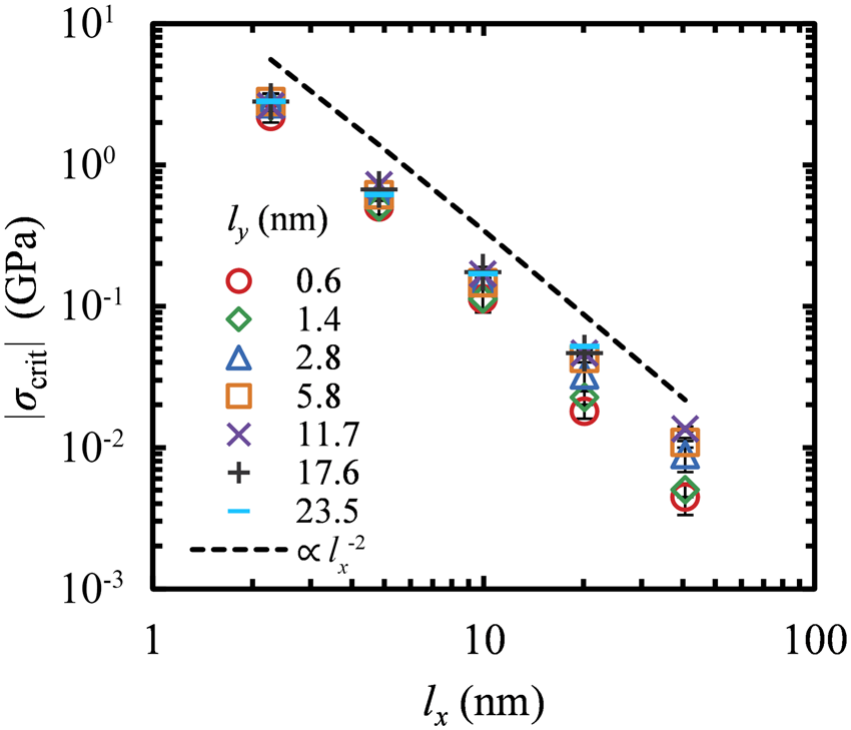
Critical buckling stress versus the length of GNR along the loading direction, *l*_*x*_, for sheets with various lateral dimensions, *l*_*y*_ = 0.6 (○), 1.4 (◊), 2.8 (△), 5.8 (□), 11.7 (×), 17.6 (+) and 23.5 (−) nm, at *T* = 300 K. The dashed line depicts a power low *σ*_crit_ ∝ *l*_*x*_^−2^. The error bars are given through *δσ*_crit_ from Eq. 2.

Figure 3 displays the length dependence of the critical buckling stress of GNRs with various widths, at room temperature. The dependence of *σ*_crit_ on the length can be described by a power law of the form:

3 $${\sigma }_{{\rm{c}}{\rm{r}}{\rm{i}}{\rm{t}}}=\frac{b}{{{l}_{x}}^{a}}$$

MD simulations on single layer graphenes with periodic edges^36–39^ predict a scaling, ${\sigma }_{{\rm{c}}{\rm{r}}{\rm{i}}{\rm{t}}}\propto {{l}_{x}}^{-2}$ (also shown in Fig. 3 by a dashed line) in agreement with the linear elasticity theory of continuum mechanics for wide slabs^46,47^.

Overall, GNRs display similar response with that of pristine graphenes, although there are subtle deviations due to the existence of the free edges. Extremely thin GNRs—which could also be regarded as nanowires—display a scaling slightly stronger than $\propto {{l}_{x}}^{-2}$. As it was discussed in the previous section, the edges of the GNR are characterized by decreased flexural rigidity and thus, the buckling resistance of GNR should decrease with decreasing widths since the ratio of the edge to inner atoms, *N*_edge_/*N*_inner_, increases. On the other hand, as the width *l*_*y*_ → ∞ the ratio *N*_edge_/*N*_inner_ → 0 and the scaling exponent goes to 2.

**Figure 4:**
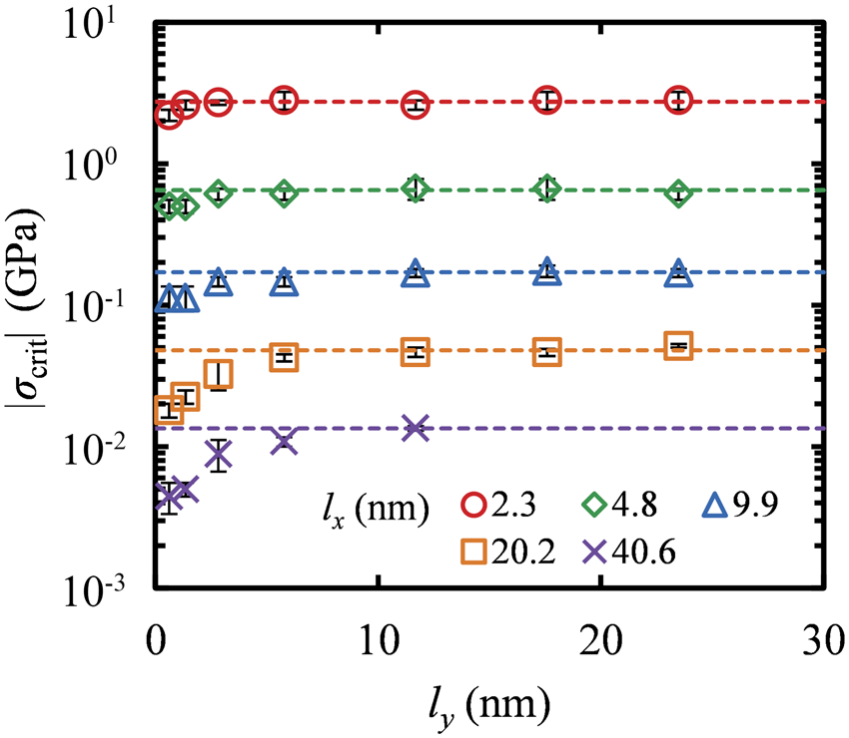
Critical buckling stress versus the width *l*_*y*_ of GNR for sheets with lengths *l*_*x*_ = 2.3 (○), 4.8 (◊), 9.9 (Δ), 20.2 (□) and 40.6 (×) nm, at *T* = 300 K. The error bars were obtained from Eq. 2. Dotted lines denote the limiting value at very large widths, ${\sigma }_{{\rm{crit}}}^{{l}_{y}\to \infty }$.

This behavior is presented clearly in Fig. 4 which depicts the width dependence of *σ*_crit_ over a broad range of length sizes. The critical buckling stress increases with increasing width and saturates to a constant value. Experimental estimates for GNRs with simply supported edges^29^ differ from the response of GNRs with free edges studied here. The former suggest that *σ*_crit_ increases as *l*_*y*_ decreases, while the latter exhibits the opposite behavior. This is due to the fact that, in GNRs with supported edges the lateral support prevents the nanoribbon from buckling^44^, therefore as the width becomes smaller the GNR become stiffer. On the other hand, a GNR with free edges is surrounded by free space and therefore, as *l*_*y*_ decreases the sheets become more flexible.

According to the continuum elasticity theory for wide plates (i.e. plates with infinite width) the critical buckling stress, ${\sigma }_{{\rm{crit}}}^{{\rm{WP}}}$, is given by the following equation:

4 $${\sigma }_{{\rm{c}}{\rm{r}}{\rm{i}}{\rm{t}}}^{{\rm{W}}{\rm{P}}}=\frac{{\pi }^{2}}{12}\frac{E}{1-{v}^{2}}\frac{{{l}_{z}}^{2}}{{{l}_{x}}^{2}/c}$$

where *E* is the elastic modulus, *ν* the Poisson ratio, and *c* a restrain coefficient which depends on the boundary conditions along the loading direction and equals to 1 or 4 for simply supported or fixed edges, respectively. In the case of plates with finite width (“plate columns”^63^ or “wide columns”^64^) the critical stress, ${\sigma }_{{\rm{crit}}}^{{\rm{WC}}}$, is modified as follows:

5 $${\sigma }_{{\rm{crit}}}^{{\rm{WC}}}=\mu {\sigma }_{{\rm{crit}}}^{{\rm{WP}}}$$

where *μ* is a coefficient depending upon the aspect ratio *R*. In the limit of zero aspect ratio, $\mu \simeq 1-{v}^{2}$ hence, Eq. 5 reduces to the Euler column buckling equation. In the limit of infinite aspect ratios, *μ* goes to unity and therefore, for all practical purposes Eq. 5 reduces to Eq. 4 for wide plates. For intermediate aspect ratios, *μ* takes values between 1 − *ν*^2^ and 1, though for the case of plate columns with fixed ends only approximate solutions can be obtained in contrary to the case of simply supported ends where exact analytical solutions exist^63^.

**Figure 5:**
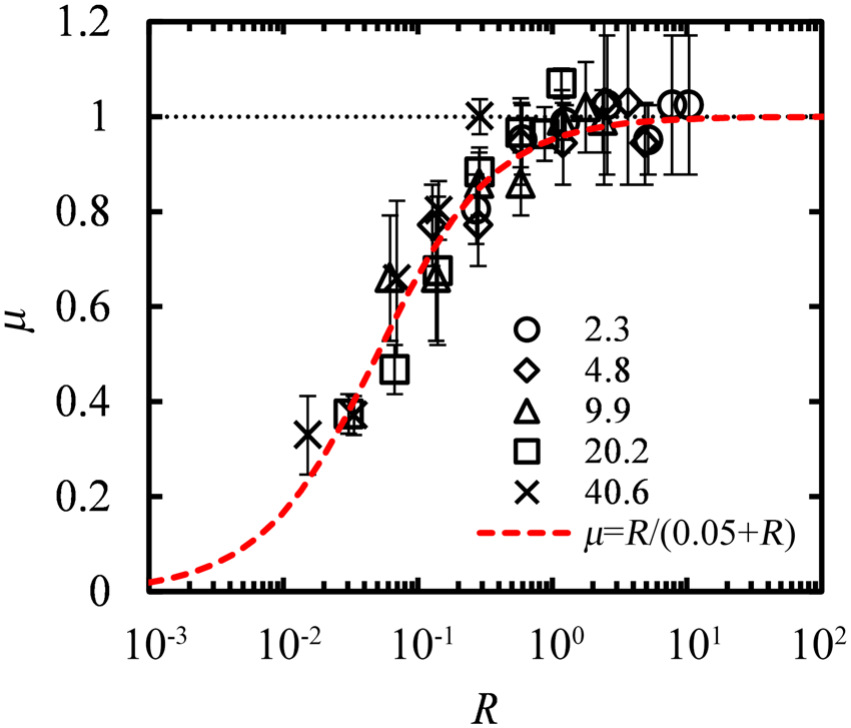
The ratio *μ* of the critical buckling stress of GNR over the corresponding value at large widths, ${\sigma }_{{\rm{crit}}}/{\sigma }_{{\rm{crit}}}^{{l}_{y}\to \infty }$ as a function of the aspect ratio *R* at *T* = 300 K. The dashed line depicts a fit over all data points with Eq. 6. Different symbols correspond to different lengths *l*_*x*_ (nm), as indicated in the figure.

Figure 5 presents a master curve made from all the data points at 300 K, in which *μ* is plotted versus *R*. The coefficient *μ* is calculated as ${\sigma }_{{\rm{crit}}}/{\sigma }_{{\rm{crit}}}^{{l}_{y}\to \infty }$, where the denominator is the critical buckling stress in the limit of infinite widths (dashed lines in Fig. 4), that scales as $\sim 1/{{l}_{x}}^{2}$. The findings for the GNRs agree qualitatively with the predictions of the wide-columns model^63^ in the sense that μ increases with increasing *R* and tends to 1 for relatively large aspect ratios (in the wide plates limit). However, there are significant discrepancies between the two models since GNRs are flexible membranes at the nanoscale and not rigid plates:

1. The wide-column model^63^ predicts $\mu \simeq 1-{v}^{2}$ as *R* → 0. This implies for GNRs that in the limit *R* → 0, *μ* is expected to be 0.96 given that *v* = 0.2 for the LCBOP force field^39^. Instead, the simulations show that *μ* is continuously decreasing with *R to much smaller values*, due to the fact that GNRs with small aspect ratios are inherently unstable and tend to collapse into more favorable configurations^40^.
2. The continuum theory^63^ predicts wide-plate behavior for *R* > 10. The simulations in GNRs show that the wide-plate behavior is reached for lower aspect ratios close to one.

The data points in Fig. 5 display a reasonable fit with the first order Hill equation^65^:

6 $$\mu =\frac{R}{0.05+R}$$

Equations 4–6 seem to approximately describe the buckling stress of all nanoribbons that we have examined at *T* = 300 K through a relation:

7 $${\sigma }_{{\rm{crit}}}=\frac{R}{0.05+R}{\sigma }_{{\rm{crit}}}^{{l}_{y}\to \infty }$$

where

8 $${\sigma }_{{\rm{c}}{\rm{r}}{\rm{i}}{\rm{t}}}^{{l}_{y}\to {\rm{\infty }}}\sim 1/{{l}_{x}}^{2}$$

### Dependence of the critical buckling stress on the chiral angle

**Figure 6:**
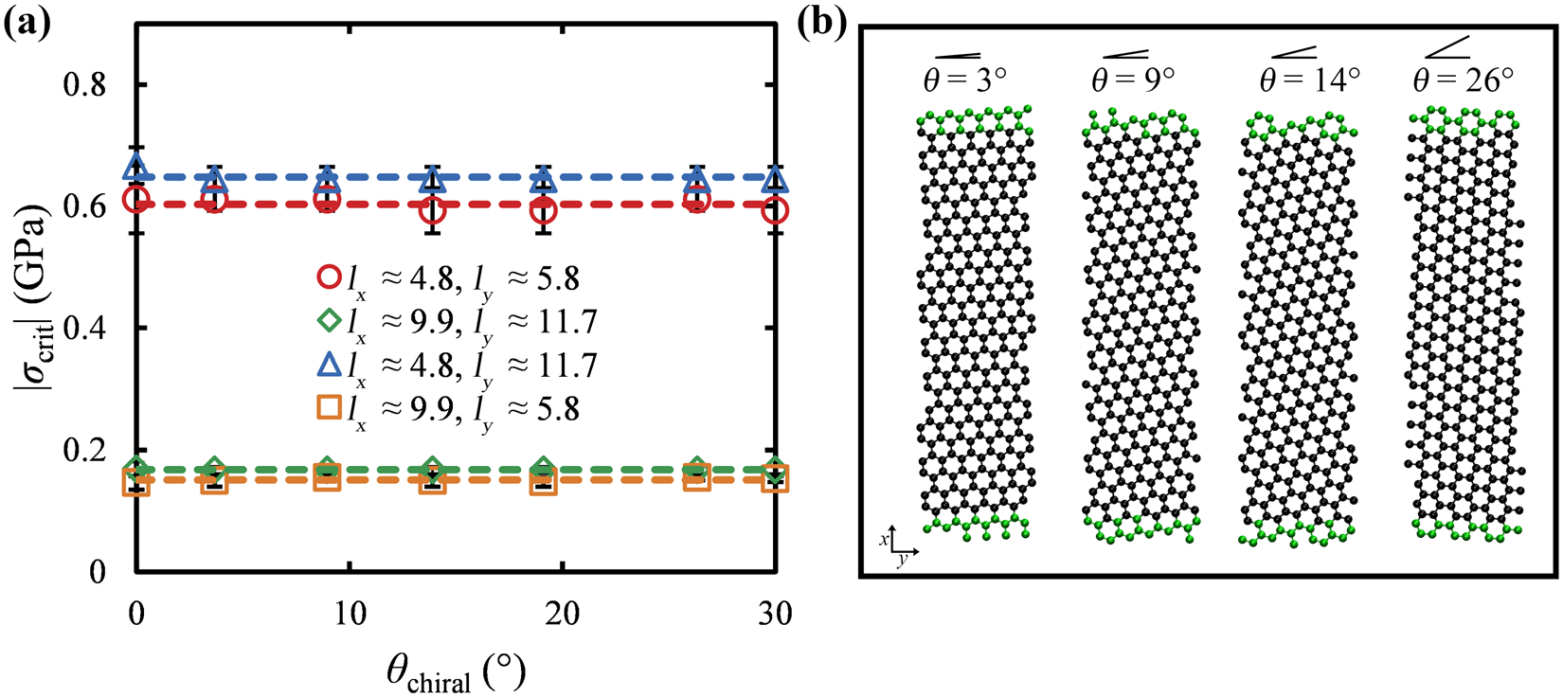
(**a**) Critical buckling stress as a function of the chiral angle along the loading direction for GNRs with variable sizes and aspect ratios. The dashed lines correspond to the average values of *σ*_crit_ over all the examined chiral angles for a particular GNR of a certain size. (**b**) Atomistic representations of GNRs oriented along various chiral angles as shown on the top of each case. The green beads display the clamped atoms wherein the compressive forces are applied, while the black beads display the inner carbon atoms.

In most practical applications, GNRs present a variety of structural imperfections at their edges such as topological defects, chiral edges, edge roughness, etc. Such structural imperfections could potentially have a noticeable effect on the mechanical properties of GNRs—and in particular—on their ability to carry compressive loads. Inevitably, GNRs with chiralities other than 0° (ZZ ends, AC edges) and 30° (AC ends, ZZ edges) are bound to possess some kind of edge roughness (see for example the right and left edges in Fig. 6b). Therefore, the dependence of the buckling resistance of GNRs on both their edge roughness and on the chiral angle^48^ along the loading direction (*θ*_chiral_) can be investigated upon examining chiral GNRs over various values of *θ*_chiral_.

Figure 6a presents the dependence of *σ*_crit_ on the chiral angle for GNRs with variable sizes and aspect ratios. The critical buckling stress seems to be insensitive to the chiral angle and coincides to the *σ*_crit_ value for *θ*_chiral_ = 0°, corresponding to the data shown in the previous sections for GNRs with applied compressive loads along the AC direction. Thus, for all practical purposes, the buckling resistance of GNRs does not display any noticeable dependence on the chiral angle as well as on small edge roughness, irrespectively of their sizes and aspect ratios. This response is also in agreement with that of wide graphenes, where the buckling resistance of periodic GNRs is independent to the chiral angle of the loading direction^36,38,39^.

### Temperature dependence of the critical buckling stress

**Figure 7:**
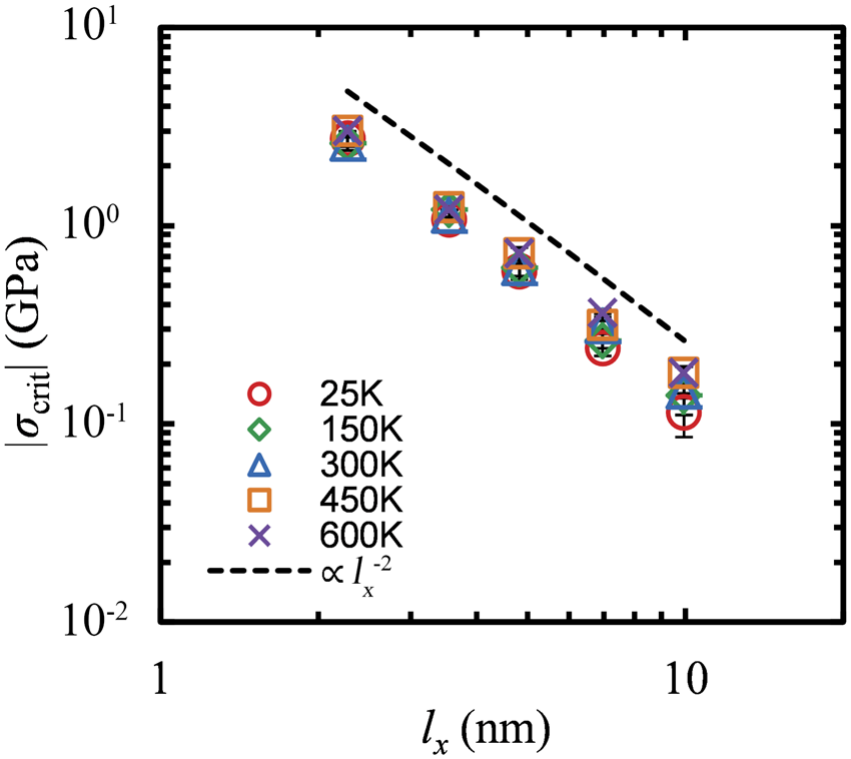
Critical buckling stress of graphene nanoribbons versus *l*_*x*_ for *T* = 25 K (○), 150 K (◊), 300 K (Δ), 450 K (□) and 600 K (×). The dashed line shows a power low *σ*_crit_ ∝ *l*_*x*_^−2^. The lateral dimensions, *l*_*y*_, for those ribbons are 5.8 nm. The error bars are equal to *δσ*_crit_ from Eq. 2.

To examine temperature effects, we show in Fig. 7 the length dependence of *σ*_crit_ for a GNR with *l*_*y*_ = 5.8 nm for temperatures up to 600 K. Overall, the entropic contribution to the critical buckling stress of GNRs is weak, due to their high stiffness, and a power law seems to describe this dependence. The GNRs display an inverse squared length dependence (the exponent of the power law in Eq. 3 is close to 2), although there is a mild increase in *σ*_crit_ with increasing temperature, especially in long GNRs.

**Figure 8:**
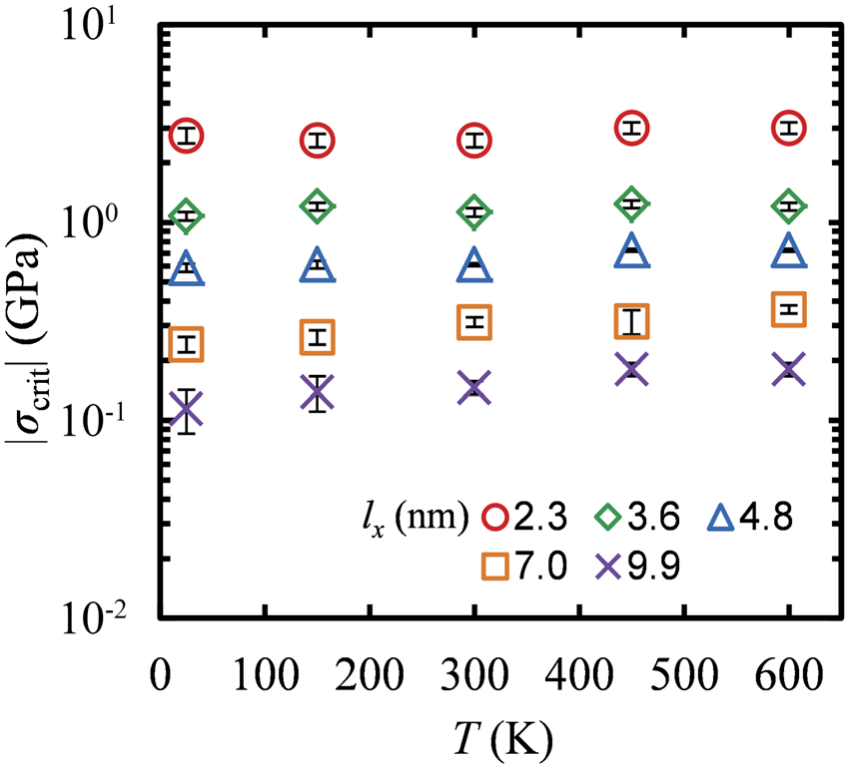
Temperature dependence of *σ*_crit_ for GNRs with width 5.8 nm and different lengths: *l*_*x*_ = 2.3 (○), 3.6 (◊), 4.8 (Δ), 7.0 (□) and 9.9 (×) nm. The error bars correspond to *δσ*_crit_ given from Eq. 2.

This can be clearly observed in Fig. 8 which shows the temperature dependence of *σ*_crit_ for GNRs with various lengths. One can see that *σ*_crit_ increases slightly with increasing temperature. This effect, on the other hand, is much less pronounced in GNRs with smaller lengths since *σ*_crit_ seems to vary with temperature within the errors given by Eq. 2.

**Figure 9:**
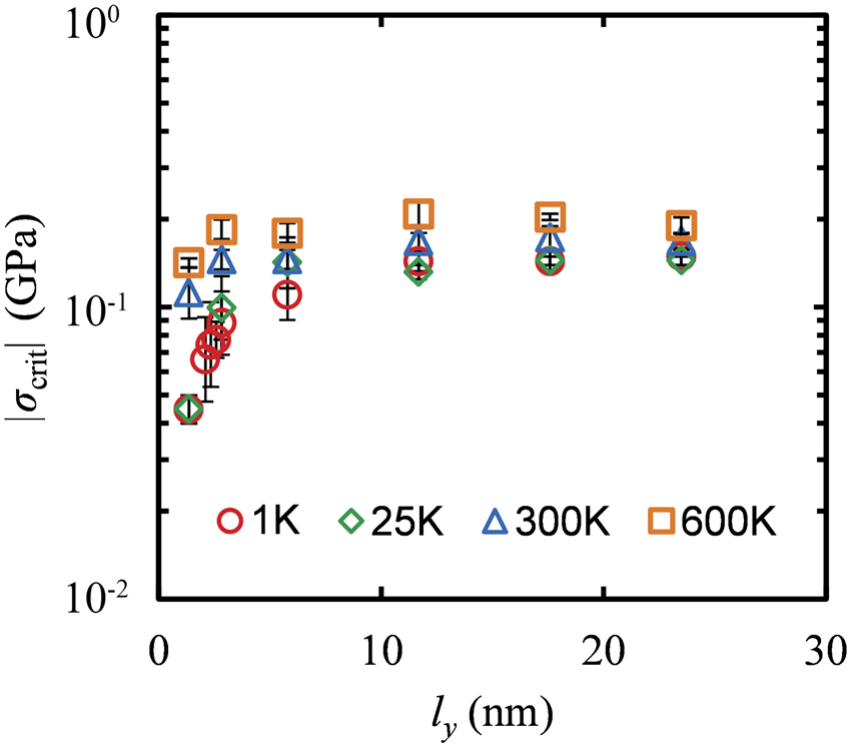
Dependence of the critical buckling stress on the nanoribbon width for *T* = 1 K (○), 25 K (◊), 300 K (Δ) and 600 K (□). The length, *l*_*x*_ of GNRs is 9.9 nm. The error bars were obtained from Eq. 2.

Figure 9 shows the width (*l*_*y*_) dependence of the critical buckling stress for GNRs with fixed length (*l*_*x*_ = 9.9 nm) at temperatures 1, 25, 300 and 600 K. It should be noted that in the case of 1 K, the GNRs were first subjected to thermal annealing from 300 to 1 K with a rate 0.003 K/fs and then they were simulated following the procedure mentioned in the methods section. This was done in order to bypass a local minima which lead to an unphysical increase of *σ*_crit_ of the order of 0.2 GPa for *l*_*y*_ = 1.4 and 2.3 nm; wider GNR’s did not display this effect. According to Fig. 9 the resistance to buckling increases with increased temperature as there is a systematic shift of the curves in Fig. 9 towards larger values of *σ*_crit_. Irrespectively of the temperature, *σ*_crit_ increases with increasing width and saturates to a constant value for larger values of *l*_*y*_, following a similar trend with that shown in Fig. 4.

**Figure 10:**
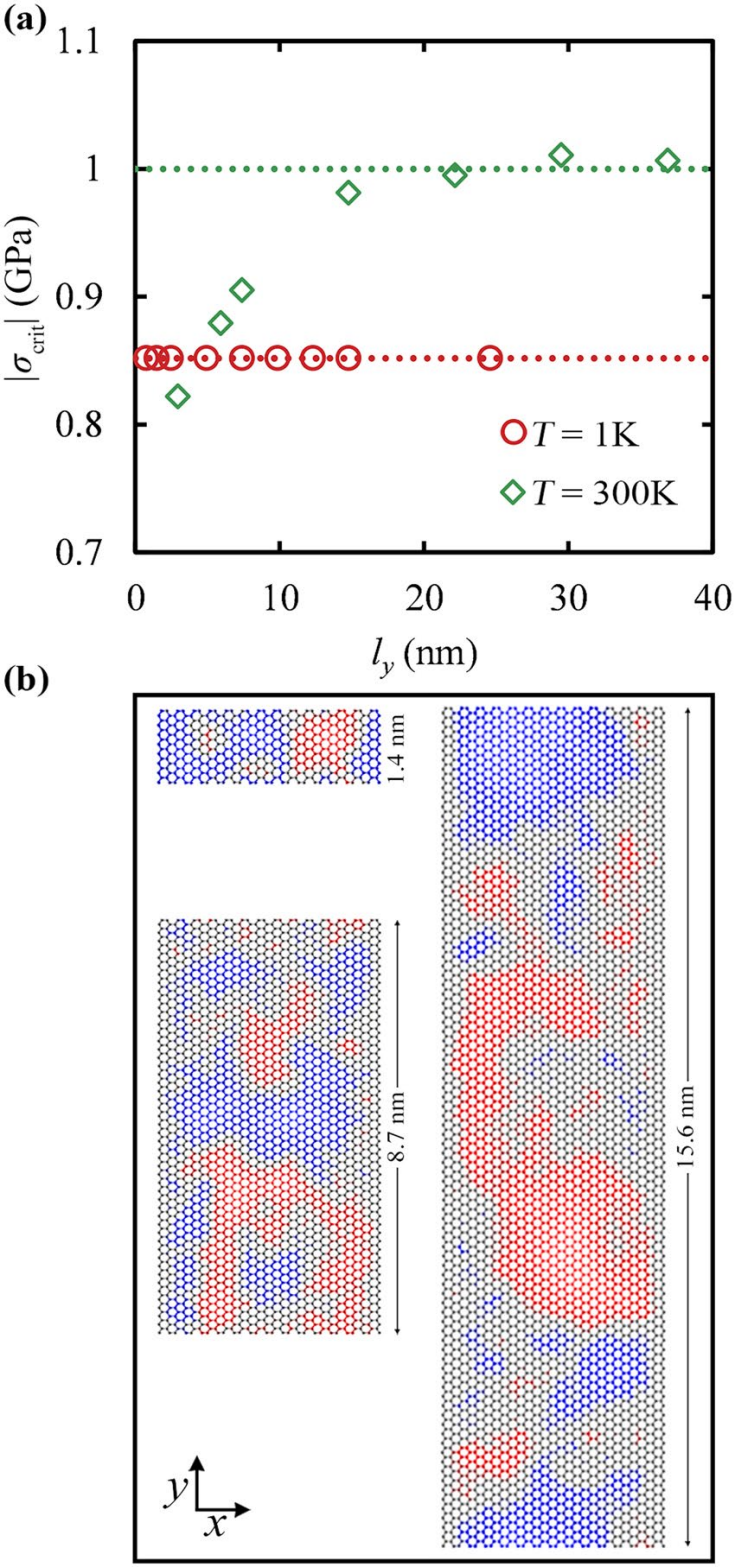
(**a**) Dependence of the critical buckling stress on the width of graphene sheets with periodic boundary conditions at the edges, for *T* = 1 K (○) and 300 K (◊). In both cases, the length *l*_*x*_ of the sheets is 4 nm. Dotted lines display the critical buckling stress in the limit of very large widths. (**b**) Atomistic representation of such graphenes with lateral dimensions equal to *l*_*y*_ = 1.4, 8.7 and 15.6 nm, at *T* = 300 K, where periodic boundary conditions are applied. Blue (red) colors illustrate positive (negative) displacements along the z-axis.

It should be noted that the increased resistance to buckling at higher temperatures is observed in graphenes which are periodic along the free edges as well. In particular, Fig. 10a presents the width dependence of *σ*_crit_ for graphene sheets with periodic boundary conditions at the edges on the lateral dimension^39^ at 1 K and 300 K. As can be seen, at *T* = 1 K the *σ*_crit_ is independent from the width of the periodic graphene since the ratio of the edge to inner atoms is by definition zero, in sharp contrast with the corresponding behavior of GNRs that showed a strong width dependence due to the out of plane displacement of their free edges (see Fig. 9). This response agrees with the predictions of the elasticity theory of wide plates in which the critical buckling stress is independent from the width^46,47,63^.

At room temperature, on the other hand, the behavior is more complicated due to the spontaneous out-of-plane fluctuations of the sheet which are not taken into account by the continuous model of wide slabs^46,47^. When the width of graphene is small, the out-of-plane fluctuations can become synchronized over the lapse of the simulation, displacing thus the majority of the atoms on the same side of the sheet (see Fig. 10b, the case for *l*_*x*_ = 1.4 nm); the instantaneous localized bending of the sheet can lead to premature buckling as seen in Fig. 10a at low *l*_*y*_. With increasing width, however, localized out-of-plane displacements can appear in different regions of the sheet at opposing directions and therefore, the buckling resistance is increased since more work is needed to bring the displacements at the same side of the sheet where the buckling is initiated. In the limit of large values of *l*_*y*_, *σ*_crit_ tends to a constant value, since the extra work required to buckle the sheet is spend to dissipate about half of the out-of-plane displacements as seen in Fig. 10b for *l*_*x*_ = 15.6 nm.

## Concluding Remarks

The behavior of graphene nanoribbons under constant compressive loads depends on their dimensions and on their aspect ratios. We have discussed in detail the corresponding stress-strain response, focusing on the quantitative dependence of the critical buckling stress.

In GNRs with low aspect ratios the buckling is initiated simultaneously along the whole end regions of the GNR, as is the case for graphenes with periodic boundary conditions in the lateral direction^39^. For large aspect ratios, the partial buckling is initiated at the corners (usually in opposing out-of-plane directions) while it is transferred to the central regions for slightly larger compressive loads.

According to the predictions of the continuum theory for wide plates^46,47^ the critical buckling stress presents an inverse squared dependence on the length for graphenes of large width; this response has been indeed observed in periodic single layer graphenes, either supported or clamped^39^. In GNRs, the length dependence of *σ*_crit_ displays a similar behavior, presenting only slight deviations due to the presence of the free edges. The free edges of a nanoribbon are more flexible than its inner regions since they are characterized by higher potential energy. Therefore, GNRs with smaller widths (large ratio of the edge to inner atoms) display reduced flexural rigidity and thus, reduced resistance to buckling. GNRs with large widths, on the other hand, present increased resistance to buckling, and eventually the critical buckling stress reaches a plateau as the lateral size continues to increase. The increase of the resistance to buckle with increasing width results from the counteraction of two different effects: (i) the ratio between the edge to the inner atoms decreases leading to increased stiffness and (ii) some additional work is required to displace the “locked” edges on the same side of the sheet which often buckle prematurely along opposing out-of-plane directions.

The behavior of the GNRs conforms to the predictions of the continuum theory for wide columns^63,64^ in the sense that for intermediate widths the resistance to buckle increases with increasing aspect ratios and saturates to a constant value in the wide plates limit. However, in the limit of small widths the response of the GNRs deviates significantly from the wide columns model, since the critical buckling strain is a continuously decreasing function of the aspect ratio, whilst the continuum model predicts a saturation to a critical buckling stress value that is 1 − *v*^2^ times smaller than the one in the limit of infinite width. The dependence of the critical buckling stress relative to its large width limit on the aspect ratio of a GNR follows a single master curve for all cases of different sizes that we have examined, at *T* = 300 K. Furthermore, the buckling resistance of chiral GNRs is insensitive to the chiral angle along the loading direction as well as to the accompanied small edge roughness.

Thermal fluctuations affect slightly the response of nanoribbons under compression, up to the maximum temperature of 600 K examined here. In GNRs the temperature dependence of *σ*_crit_ should not be attributed entirely to the behavior of the free edges since simulations on periodic graphenes showed increased buckling resistance with increased temperature as well. The reasoning behind this increased critical buckling can be attributed to the extra work required to overcome the out-of-plane displacements in different directions in order to initiate the buckling on a particular side of the sheet.

## Methods

**Figure 11:**
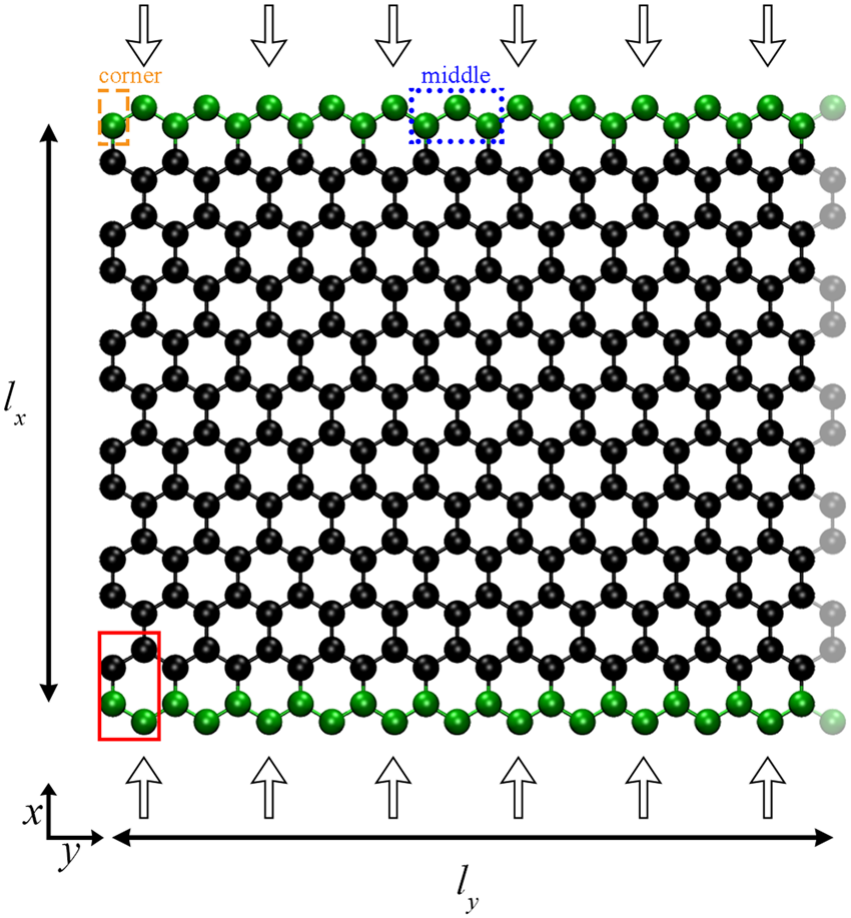
Atomistic representation of a graphene ribbon with “clamped” ends (green beads) under uniaxial compressive loads. This configuration was generated upon replicating the 4-atom cell (surrounded by the red rectangle), *n*^AC^ = 6 and *n*^ZZ^ = 12 times along the *x* (armchair) and *y* (zigzag) direction, respectively; while removing the rightmost line of atoms (opaque beads). The displacement of the atoms lying in the fixed region (green beads) is constrained on the *xy* plane, while the displacement of those at the center of the clamped region (surrounded the blue box) is constrained to be along the *x*-direction. The orange box depicts one of the GNR’s corner.

To design rectangular GNRs that we discuss here, the 4-atom cell shown in Fig. 11 (atoms surrounded by the red rectangle) was replicated *n*^ZZ^ and *n*^AC^ times along the zigzag and the armchair direction, respectively, while removing the rightmost line of atoms to make the ribbon symmetric. The distance between neighboring carbon atoms equals to *d*_C−C_ = 0.142 nm.

The length of the ribbon along the loading direction (*l*_*x*_) equals to the distance between the clamped regions (GNR ends), while its width, i.e. the size of the flake along the lateral dimension (the distance between the GNR edges) is either ${l}_{y}^{{\rm{ZZ}}}=\sqrt{3}{d}_{C-C}({n}^{{\rm{ZZ}}}-0.5)$ or ${l}_{y}^{{\rm{AC}}}=3{d}_{C-C}{n}^{{\rm{AC}}}$ depending on whether the transverse direction has zigzag (as in the case shown in Fig. 11) or armchair ends, respectively.

In order to design chiral GNRs the following procedure was followed: initially, we designed GNRs with very large dimensions; then, these GNRs were rotated along a specific chiral angle^48^ with respect to the loading direction; an orthogonal region with the desired dimensions of the final GNR was defined; finally, all the atoms lying outside this region were removed thus, the remaining carbon atoms form chiral GNRs such as those depicted in Fig. 6b. The dimensions of orthogonal GNRs with intermediate chiral angles, 0° < *θ*_chiral_ < 30° such as those shown in Fig. 6b cannot be defined with the same rigor as in GNRs with AC and ZZ edges, thus they are assumed to be equal to the dimensions of the orthogonal region used to cut the chiral GNRs.

The depth of the clamped regions does not affect the response of the GNR as long as its length *l*_*x*_ (as defined above) and the total imposed force remains constant^39^. The simulated systems are non-periodic in every direction, i.e., the GNRs are centralized to the center of the simulation box, being surrounded by vacuum.

The loading of graphene sheets was performed by applying constant compressive forces at the atoms lying in the opposite clamped regions (green beads in the schematic of Fig. 11). The magnitude of the force per-atom was set to:

9 $${F}_{{\rm{per}}{\rm{atom}}}=\frac{\sigma {l}_{y}{l}_{z}}{{n}_{{\rm{atoms}}{\rm{per}}{\rm{end}}}}$$

where *σ* is the target compressive stress, *l*_*z*_ is the thickness of a graphene sheet which was considered equal to the interlayer distance in graphite (0.335 nm)^66,67^ and *n*_atoms per end_ is the number of atoms at each GNR’s end.

Unconstrained GNRs constitute highly unstable systems when subjected to uniaxial compressive loads, since they tend to rotate along the *y* and *z* axes^39,40^. One has to apply geometric constrains to prevent such rotations. The type of the applied constrains affects in a great deal the mechanical response of slabs^46,47^. The main geometric constrains used to stabilize such systems are the incorporation of simply supported and fixed ends^46,47^; the latter was employed in the current study. In particular, to prevent the rotation of the sheets along *y-*axis, the displacements of the atoms belonging to the clamped region (green beads in Fig. 11) are constrained on the *xy* plane. To prevent rotations along *z*-axis, the three atoms lying to the center of the clamped region (atoms surrounded by the dotted rectangle in Fig. 11) can only move along the loading direction. Thus, the atoms in the GNR ends can freely move along the loading direction and transfer the stress to the interior of the material, while almost all of them (apart from the three central atoms) can also move to the lateral direction in order to capture volume conserving effects related to Poison’s ratio.

Our molecular dynamics (MD) simulations were performed using the Large-Scale Atomic-Molecular Massively Parallel Simulator (LAMMPS) package^58^. The interactions between carbon-carbon atoms are described by the LCBOP force field^59^. It has been shown^39^ that this potential produces a qualitatively similar compressive behavior of graphenes as other relevant force fields^57,60,61,68^. LCBOP also provides accurate phonon dispersion curves for single layer graphenes^69^. The integration of the equations of motion was performed with the velocity-Verlet algorithm^70^ using a time step of 1 fs. The temperature of the systems was maintained at its desired value upon incorporating a Nosé-Hoover thermostat^71,72^ with the MTK correction^73^ and an effective relaxation time of 0.1 ps. Each GNR was simulated for 10 ns free from any loads at a given temperature, in order to estimate its reference dimensions from which the strains are computed when stress is applied.

A typical constant stress MD simulation comprises the following phases:

1. Equilibration of the graphene sheet, free from any loads, at the desired temperature for 1.0 ns.
2. Simulation of the GNRs under constant compressive loads for up to 5 ns to extract the stress-strain curves. The dimensions of the GNR were being recorded every 100 time steps for the computation of the time-averaged strain in a post-processing stage. It should be noted that in case that the GNR buckles then only the strain values during the post buckling phase are taken into account.

Furthermore, additional simulations for up to 50 ns were conducted for stress values near the critical transition regime in order to minimize possible relaxation effects.

MD simulations to obtain compressive stress-strain curves were performed over a broad range of temperatures (25–600 K) and aspect ratios, *R* = *l*_*y*_/*l*_*x*_, ranging from ~0.01 to ~10. The minimum (maximum) length of the studied sheets along the *x*-axis is about 2 nm (40 nm); while the minimum (maximum) width along the *y*-axis is about 0.6 nm (23 nm). The behavior of periodic graphenes under compression has been shown to be insensitive to the chiral angle of the loading direction^36,38,39^. Unless mentioned otherwise, the results presented consider compressive loads along the AC direction (see Fig. 11). A few test simulations with loads along the ZZ direction displayed near identical behavior concerning the length dependence of the critical buckling stress (see Fig. S1 in the supporting information), confirming the above mentioned insensitivity of the compressive behavior on the loading direction. Results concerning intermediate chiral angles 0° < *θ*_chiral_ < 30° are presented in the Results section of the manuscript.

The datasets generated during and/or analyzed during the current study are available from the corresponding author on reasonable request.

## Electronic supplementary material

Supplementary Information Section
