# Supplementary material for: Compressive response and buckling of graphene nanoribbons
Source: Sci Rep. 2018 Jun 25;8:9593. doi: 10.1038/s41598-018-27808-0 (PMC6018628; doi:10.1038/s41598-018-27808-0)
Supplement: Supplementary file 1 — Supplementary Information Section [file 41598_2018_27808_MOESM1_ESM.pdf]

Compressive response and buckling of graphene nanoribbons

A. P. Sgouros[1], G. Kalosakas[2, 3, 4*], K. Papagelis[2, 5] and C. Galiotis[2, 6]

[1]*School of Chemical Engineering, National Technical University of Athens (NTUA), GR-15780 Athens, Greece.*

[2]*Institute of Chemical Engineering Sciences - Foundation of Research and Technology Hellas (FORTH / ICE-HT), GR-26504 Patras, Greece*

[3]*Department of Materials Science, University of Patras, GR-26504 Patras, Greece*

[4]*Crete Center for Quantum Complexity and Nanotechnology (CCQCN), Physics Department, University of Crete GR-71003 Heraklion, Greece*

[5]*Physics Department, University of Patras, GR-26504 Patras, Greece*

[6]*School of Chemical Engineering, University of Patras, GR-26504 Patras, Greece*

**Corresponding author*. E-mail: georgek@upatras.gr

S1. Critical buckling stress along AC and ZZ chiral loading directions.

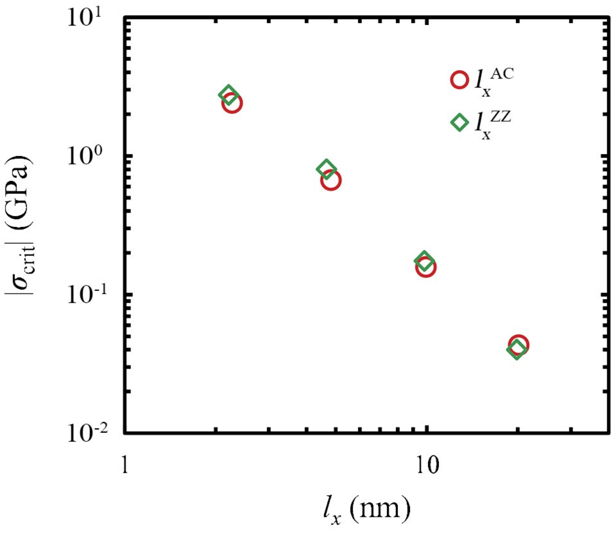

Figure S1. Critical buckling stress versus the length of the AC (circles) and ZZ (diamonds) loading direction.

S2. Minimized configurations of GNR with variable aspect ratios.

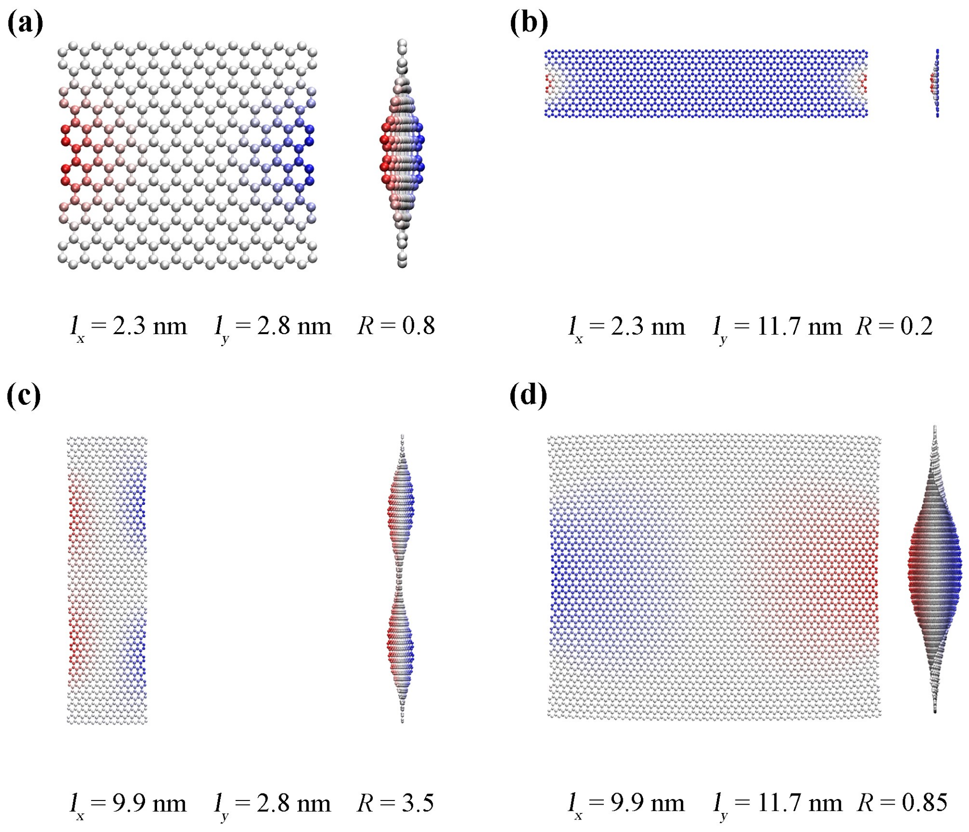

Figure S2. The minimized configurations of GNR of various aspect ratios with fixed zigzag and free armchair edges, modeled by the LCBOP[57] force field.

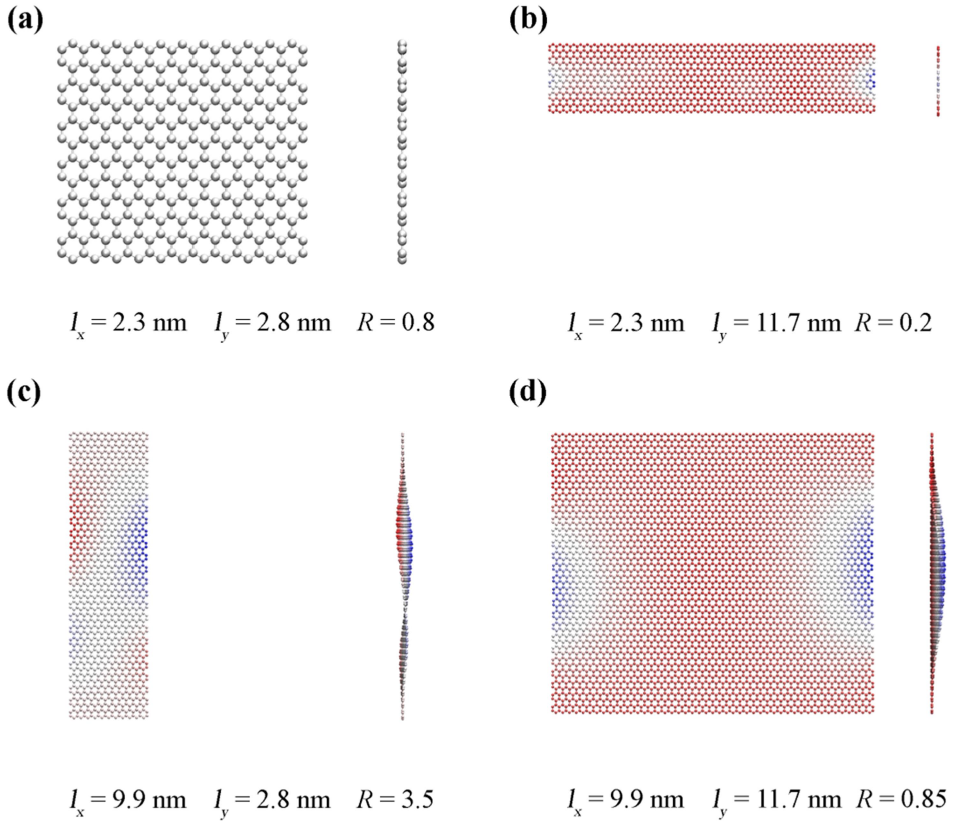

Figure S3. The minimized configurations of GNR of various aspect ratios with fixed zigzag and free armchair edges, modeled by the TERSOFF[58,59] force field.

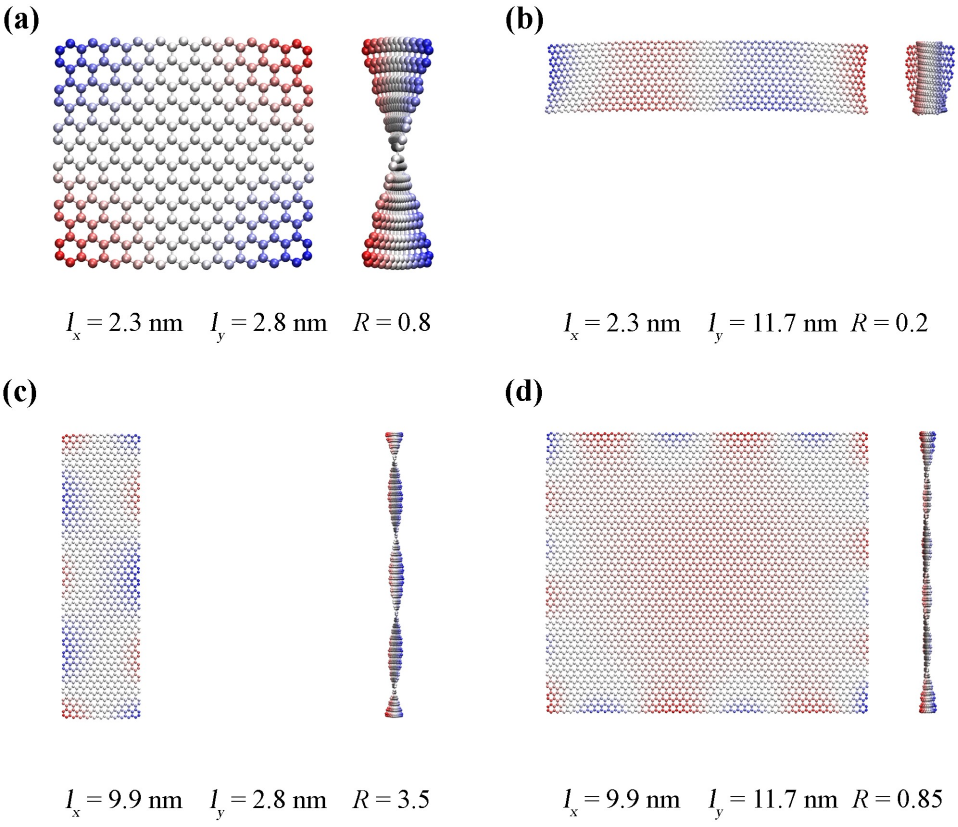

Figure S4. The minimized configurations of GNR of various aspect ratios with free edges modeled by the LCBOP[57] force field.

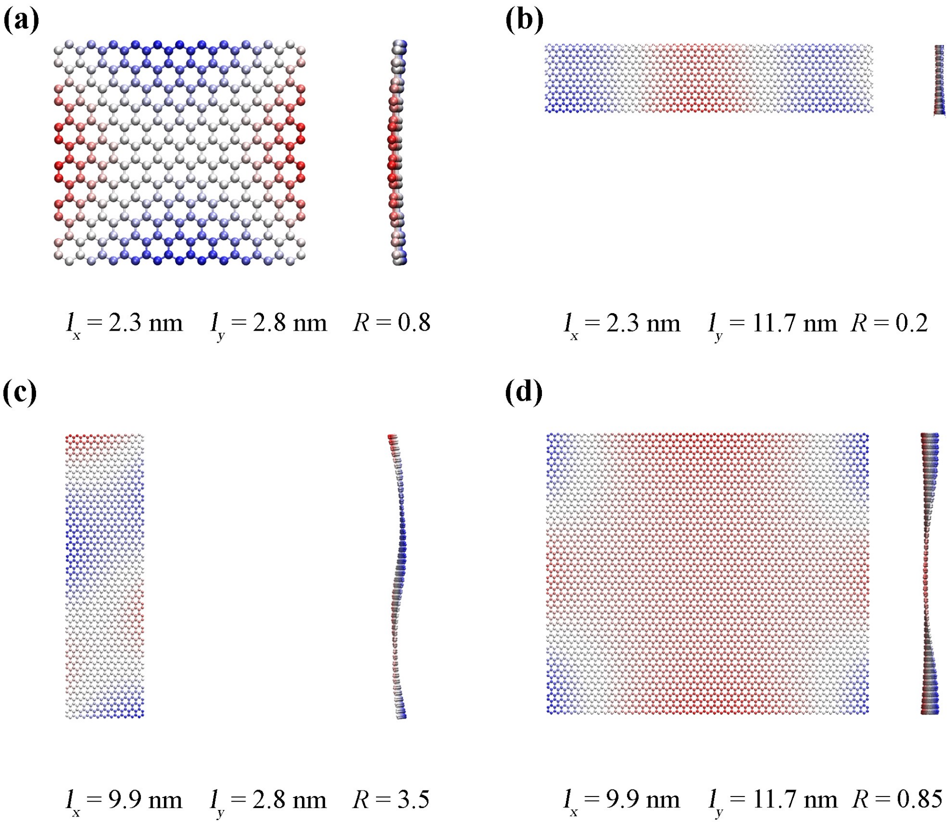

Figure S5. The minimized configurations of GNR of various aspect ratios with free edges modeled by the TERSOFF[58,59] force field.
